# Mechanochemical Synthesis of γ‑Fe_2_O_3_ Nanoparticles@N-Doped Mesoporous Carbons Derived from Biomass for Oxygen Reduction

**DOI:** 10.1021/acsomega.6c05376

**Published:** 2026-08-10

**Authors:** Hung-Ta Yu, Ling-Wei Wei, Shou-Heng Liu

**Affiliations:** Department of Environmental Engineering, 34912 National Cheng Kung University, Tainan 70101, Taiwan

## Abstract

A facile synthesis method via the ball-milling coupled with simultaneous carbonization and activation with KOH at different temperatures was employed to prepare γ-Fe_2_O_3_ nanoparticles-supported N-doped mesoporous carbons (denoted as γ-Fe_2_O_3_@N-doped C_X_-*T*) from a variety of biomass raw materials (sugar cane bagasse, coffee grounds, and luffa) as precursors. The obtained γ-Fe_2_O_3_@N-doped C_X_-*T* catalysts are characterized by using various spectroscopic measurements (i.e., X-ray diffraction (XRD), transmission electron microscopy (TEM), and X-ray photoelectron spectroscopy (XPS)) and tested for oxygen reduction reaction (ORR) in an alkaline solution via cyclic and liner sweep voltammetry. As a result, the γ-Fe_2_O_3_@N-doped C_S_-600 catalyst prepared from sugar cane bagasse at 600 °C exhibits the superior catalytic performance toward ORR with positive onset potential (1.01 V vs reversible hydrogen electrode), 4-electron transfer pathway, methanol-resistance, and operation stability, which is comparable to a commercially obtainable Pt/C catalyst. XPS analysis reveals that the nitrogen-doped carbons, i.e., pyridinic-N and graphitic-N, are the active species of catalysts for ORR. XRD and TEM images show that the formation of γ-Fe_2_O_3_ on N-doped mesoporous carbons may also be the cocatalysts for boosting ORR performance. This study not only proposes a promising candidate as a nonprecious ORR cathode for alkaline fuel cells but also realizes the purpose of resource recovery and utilization.

## Graphical abstract

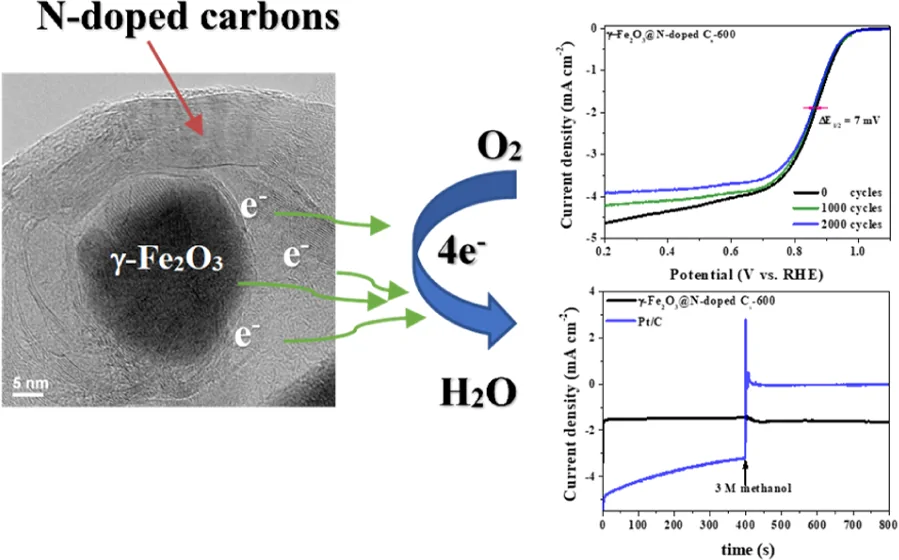

## Introduction

Fuel cells are regarded as clean energy devices for automobile and stationary applications because of their superior energy efficiency, surpassing power density, and zero CO_2_ emissions.^1,2^ Nonetheless, the slow kinetics of the oxygen reduction reaction (ORR) in the cathode is a vital issue to be addressed in the progress of fuel cells. Up to now, noble platinum (Pt) and Pt-based materials have been generally considered as the utmost active and effective electrocatalysts for ORR. Nevertheless, the disadvantages of high cost, scarcity, and worse durability greatly obstructed the large-scale commercialization of fuel cells.^3,4^ Great efforts have been made in exploring the inexpensive and more active nonprecious metal electrocatalysts to replace Pt-based catalysts. For example, the cathodes fabricated from transition-metal macrocyclic compounds, transition metal chalcogenides, and non-noble metal-modified carbons have been recognized as the feasible options toward fuel cell applications.^5^

Recent research demonstrates that heteroatoms (including N, S, B, P, F, etc.) incorporated into carbons, e.g., fullerene,^6^ nanofibers,^7^ graphene,^8^ and amorphous carbons,^9^ possess superior ORR performance in an alkaline solution. Among these chemical dopings with nonmetal atoms, nitrogen is considered to be one of the outstanding elements owing to its similar atomic size and strong valence bonds with carbon atoms.^10^ The improved ORR behavior of nitrogen-doped carbon may be due to the modification of the electronic structure, resulting in the activation of π electrons in the carbon via the conjugation with the lone-pair electrons of N atoms. Nitrogen atoms can offer electrons to the conjugated π orbital causing electropositivity of adjacent carbon atoms, readily facilitating the O_2_ adsorption and carry out the splitting of the O–O bond.^11–13^ In addition, the carbon-supported transition metal–N composites (i.e., M–N/C, M = Fe, Co, etc.) were reported to be effective non-noble metal ORR catalysts which were potential for replacing the noble-based catalysts in the field of alkaline fuel cells.^14–16^ In terms of these M–N/C electrocatalysts, the Fe–N/C catalysts gained much attention as ORR catalysts due to their superb electrocatalytic properties (i.e., low cost and good activity). In general, the Fe–N/C catalysts with comparable ORR activity as compared to that of commercial Pt/C were fabricated by carbonizing the mixture of carbon, nitrogen, and iron metal precursors under inert or N-containing gas.^16–18^ In particular, the Fe_2_O_3_ nanoparticles supported on N-doped carbons were reported to be one of the promising electrocatalysts for ORR.^19–21^

It has been revealed that the porous carbon materials can serve as an important part in the augmentation of ORR catalytic performance (i.e., activity and durability).^22–24^ Generally, carbon blacks/activated carbons, which were derived from expensive and nonrenewable resources such as petroleum residues, wood, coal, peat, and lignite,^25^ were commonly used as catalyst supports in the fuel cells. Therefore, in the past few years, research works have been concentrated on the synthesis of hierarchically porous carbons based on biomass and agriculture wastes,^26,27^ such as rice husk, corn cob, olive stones, waste coffee beans, wood, bamboo, sugar cane bagasse, and so on.

Recently, numerous synthetic strategies^28^ have been developed to fabricate heteroatom-doped carbon-based ORR electrocatalysts, including hydrothermal synthesis, template-assisted carbonization, molten salt methods, solution plasma synthesis, and mechanochemical approaches. Among these, solution plasma synthesis^29^ has attracted considerable attention because it enables the rapid, one-step fabrication of metal/carbon composite catalysts under relatively mild conditions without prolonged thermal treatment. Nevertheless, plasma-assisted techniques generally require specialized plasma equipment and face challenges in the large-scale utilization of biomass feedstocks. Ball milling is a simple and efficient method for particle size reduction and material mixing through impact and friction, which can induce bond breakage, increase surface area, and generate reactive sites that facilitate solid–solid or solid–liquid reactions.^30^ Structural analyses (e.g., X-ray and Raman) revealed increased defects and tunable material properties after milling.^31^ Ball milling can be performed under dry or wet conditions with key parameters including mill type, rotation speed, ball size, and environmental conditions. Overall, ball-milling is a versatile and scalable technique for material synthesis and modification.

In this work, a synthesis route was presented to preparing Fe–N/C materials from various biomass materials with different carbonized temperatures. The resultant biocarbons with a N-doped mesoporous structure and a highly graphitized framework were used as the support to host γ-Fe_2_O_3_ nanoparticle electrocatalysts (denoted as γ-Fe_2_O_3_@N-doped C_X_-*T*) via a ball-milling process. This mechanochemical pretreatment combined with KOH activation provides a solvent-minimized and scalable strategy to convert renewable biomass into hierarchically porous N-doped carbons with abundant accessible active sites. The analysis of various spectroscopies (i.e., X-ray diffraction (XRD) and X-ray photoelectron spectroscopy (XPS)), transmission electron microscopy (TEM), and nitrogen adsorption–desorption isotherms) was carried out to study the physicochemical properties of γ-Fe_2_O_3_@N-doped C_X_-*T*. The electrochemical ORR was also studied on γ-Fe_2_O_3_@N-doped C_X_-*T* catalysts in a base solution via linear sweep voltammetry and rotating disk electrode (RDE) technique. Electrochemical analysis data demonstrated that γ-Fe_2_O_3_@N-doped C_S_-600 exhibits the better ORR performance with the higher positive onset potential and current density which may be a prospective cathode for fuel cells.

## Experimental Section

### Preparation of Carbon Supports

To improve precursor homogeneity (remove water-soluble impurities and extractives) and facilitate pore development during pyrolysis, raw materials (sugar cane bagasse, coffee grounds, and luffa) were soaked in boiling water at 100 °C for 1 h. Then, the washed samples were subsequently dried at 100 °C for 12 h to eliminate residual moisture and ensure reproducible carbonization conditions (Scheme 1). The resultant samples were then ground by a rotary-type grinding machine, followed by carbonizing at 350 °C for 15 min under atmospheric conditions. About 2 g of biocarbon was soaked with potassium hydroxide (KOH, Sigma-Aldrich, >85%) (KOH (g)/char (g) = 4:1). The resultant solution was further dried overnight at 100 °C, then pyrolyzed in a furnace at a heating rate of 5 °C min^–1^ to 900 °C, and kept at that temperature for another 1 h under the flow of N_2_ gas. The obtained samples were rinsed with 0.2 M HCl and deionized (DI) water, filtered, and dried for 12 h.

**Scheme 1:**
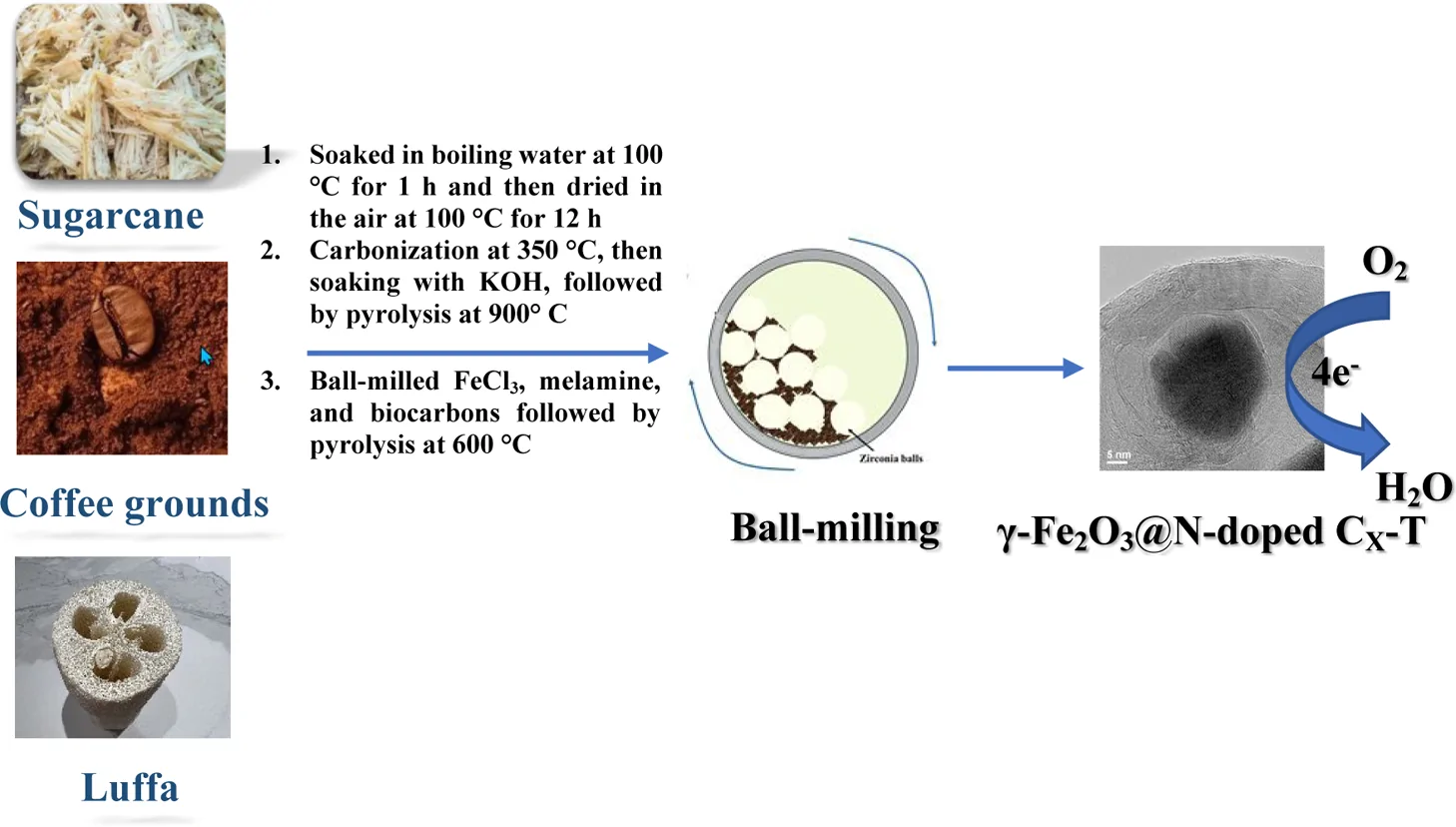
Preparation Procedures for γ-Fe_2_O_3_@N-doped C_X_-*T*

### Preparation of γ-Fe_2_O_3_@N-Doped C_X_-*T* Electrocatalysts

The Fe–N/C biocarbons were synthesized by the ball-milling technique. Ferrous chloride (ACROS, 98%) melamine (Alfa Caesar, 98%) and biocarbons (total weight of 2.2 g) were employed as the iron precursor, nitrogen precursor, and carbon source, respectively. The powder and grinding balls (5 mm ZrO_2_ balls) were poured into a 50 mL grinding jar in a planetary ball milling machine (PM 100, Retsch Inc., Germany), followed by ball-milling at a rotation rate of 400 rpm for 6 h. The milled powder was centrifuged with DI water, removed from the upper liquid, and dried in an oven at 60 °C overnight. Then, the resulting materials were pyrolyzed in a tubular furnace under a N_2_ atmosphere with a heating rate of 5 °C min^–1^ to various temperatures (*T* = 500, 600, and 700 °C). The attained samples prepared from coffee grounds as a carbon precursor were denoted as γ-Fe_2_O_3_@N-doped C_C_-*T* (*T* = 500, 600, and 700 °C). Likewise, different carbon precursors (sugar cane bagasse, coffee grounds, and luffa) were used to prepare Fe–N/C at 600 °C, which were denoted as γ-Fe_2_O_3_@N-doped C_S_-600, γ-Fe_2_O_3_@N-doped C_C_-600, and γ-Fe_2_O_3_@N-doped C_L_-600, respectively.

### Catalyst Characterizations

The crystallization structure of all catalysts was analyzed by XRD via a PANalytical (X’Pert PRO) diffractometer. The morphology of the obtained samples was examined by using TEM (JEOL JEM-1400). The chemical speciations and structure of catalysts were inspected by XPS (PHI 5000 VersaProbe). The porosities of catalysts were investigated by a nitrogen adsorption analyzer (Micromeritics ASAP 2020) at 77 K. The contents of Fe in the samples were measured by inductively coupled plasma optical emission spectrometry (ICP-OES, Horiba Jobin Yvon ULTIMA 2000).

### Electrochemical Measurements

The electrocatalytic activity of various samples was carried out by using a galvanostat/potentiostat (Autolab, PGSTAT30) via cyclic voltammetry (CV) and linear sweep voltammetry (LSV) techniques. A glassy carbon (GC) electrode (diameter = 5 mm), a Ag/AgCl electrode, and a Pt wire were employed as the working, reference, and counter electrodes, respectively. The working electrode was done by the subsequent procedures: 5 mg of the obtained catalyst was dispersed ultrasonically in a 2.5 mL mixture of DI water and Nafion (Aldrich, 5.0 wt %) with a volume ratio of 97.5/2.5 for 60 min. Subsequently, 15 μL of the prepared ink was deposited onto the GC electrode (0.15 mg_catalyst_ cm^–2^) and dried at 25 °C in the air for 1 h. The ORR performance (activity, methanol tolerance, and durability) was evaluated in an O_2_-saturated 0.1 M KOH with the scan and rotation rates of 5 mV s^–1^ and 1600 rpm, respectively, in the potential range of 0.2–1.1 V vs reversible hydrogen electrode (RHE). RDE analysis at various rotation rates was employed to measure the ORR kinetics and calculate the electron transfer number (*n*) during ORR. The *n* values and limiting current were evaluated by using the Koutecky–Levich (K–L) equation (eqs 1 and 2).

1 $$1/j=1/j_{K}+1/j_{D}=1/j_{K}+1/B\omega^{1/2}$$

2 $$B=0.62nFC_{0}D_{0}^{2/3}\nu^{-1/6}$$

where *j*_K_ and *j*_D_ represent the kinetic and diffusion current density, respectively. ω signifies the angular velocity of disk. The *n* value indicates the overall electron transferred number during ORR. *F* is the Faraday constant (i.e., 96,485 C mol^–1^). *C*_0_ and *D*_0_ are the concentration of O_2_ (i.e., 1.2 × 10^–6^ mol L^–1^) and the diffusion coefficient of O_2_ (i.e., *D*_0_ = 1.9 × 10^–5^ cm^2^ s^–1^), respectively. ν represents the kinetic viscosity of the electrolyte (i.e., ν = 0.01 cm^2^ s^–1^).

In addition, the rotating ring disk electrode (RRDE) was used to measure the *n* values by using the aforementioned Autolab (PGSTAT30) workstation. Accordingly, the *n* and the percentage of H_2_O_2_ (%) were further calculated by the following formula (eqs 3 and 4):

3 $$n=4\times \frac{I_{d}}{I_{d}+I_{r}/N}$$

4 $$H_{2}O_{2}\;\left(\%\right)=100\times \frac{2I_{r}/N}{I_{d}+I_{r}/N}$$

where *I*_r_ represents the ring current, *I*_d_ denotes the disk current, and *N* is the current collection efficiency.

## Results and Discussion

The CV curves of the prepared γ-Fe_2_O_3_@N-doped C_C_-*T* catalysts are shown in Figure 1a. Among γ-Fe_2_O_3_@N-doped C_C_-*T*, γ-Fe_2_O_3_@N-doped C_C_-600 has the highest specific capacitance of 0.305 F g^–1^. To investigate the ORR activity of the catalysts, LSV tests were carried out under an oxygen-saturated 0.1 M KOH solution. As shown in Figure 1b, the γ-Fe_2_O_3_@N-doped C_C_-600 samples exhibit the superior activity (onset potential (*E*_onset_) = ca. 0.91 V vs RHE, limiting current density (3.80 mA cm^–2^), and half-wave potential ((*E*_1/2_) = ca. 0.78 V vs RHE)). Moreover, the Tafel slopes (see Figure 1c) in the mixed control region are 137, 109, and 102 mV dec^–1^ for γ-Fe_2_O_3_@N-doped C_C_-500, γ-Fe_2_O_3_@N-doped C_C_-700, and γ-Fe_2_O_3_@N-doped C_C_-600, respectively. This result indicated that γ-Fe_2_O_3_@N-doped C_C_-600 exhibited a high charge-transfer and favorable reaction kinetics. The number of transferred electrons (*n*) and the percentage of hydrogen peroxide generated during ORR were examined by using RRDE in Figure 1d. The results indicate that more than 3.97 electron transfer observed for three catalysts at various potentials between 0.2 and 0.6 V, indicating that the 4-electron pathway dominates the ORR. Additionally, the percentage of hydrogen peroxide generated is found to be very low. In addition, the *n* values of ORR are obtained from the K–L plots in the potential of 0.4–0.6 V vs RHE, as shown in Figure 1e–g. As a result, the similar *n* values obtained by using RDE and RRDE are observed. Furthermore, the chronoamperometric curves of γ-Fe_2_O_3_@N-doped C_C_-600 are much stable after 10,000 s of chronoamperometric measurements and still maintain 80% of normalized current, as shown in Figure 1h. Furthermore, methanol tolerance tests were performed by dosing 1.0 M of methanol. As a result, the LSV curves could not be notably effected in the presence of methanol, as observed in Figure 1i.

**Figure 1:**
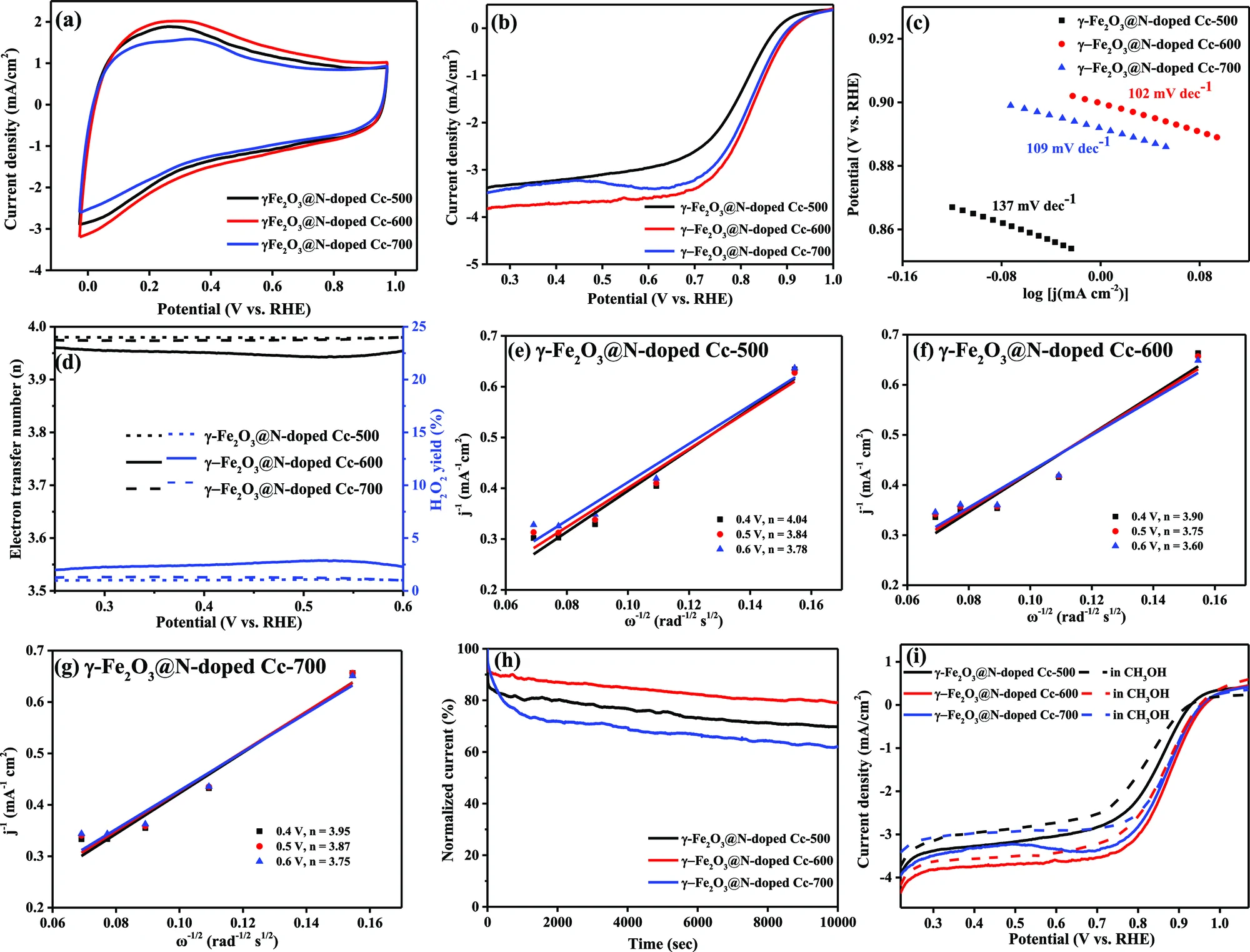
(a) CV, (b) LSV, (c) Tafel plots, (d) H_2_O_2_ yield (%) and electron transfer number, (e–g) K–L plots, (h) current–time chronoamperometric response, and (i) polarization curves (with 1 M methanol) of γ-Fe_2_O_3_@N-doped C_C_-*T*.

The effect of raw biomasses on the preparation of γ-Fe_2_O_3_@N-doped mesoporous carbons and their corresponding ORR performance is further studied. Generally, biomasses contain different compositions and physicochemical properties. The coffee grounds contain lignocellulosic materials with a high content of oil (10–15 wt %). Sugar cane bagasse has great amounts of lignocelluloses, i.e., cellulose, hemicellulose, and lignin. Luffa sponge, a fiber-connecting porous natural plant, is hollow with parallel micrometer-scale multichannels. These biomasses are used as the precursors for the fabrication of hierarchically mesoporous biocarbons with hierarchical pore networks and a complex micro/macrostructure. Such compositional and structural differences can influence the graphitization degree, pore architecture, and dispersion of γ-Fe_2_O_3_ nanoparticles, thus affecting mass transport, active-site exposure, and ultimately the ORR catalytic performance. It should be noted that natural biomass precursors inherently contain trace amounts of inorganic minerals, such as silica, alkali metals, and alkaline earth metals which could adversely affect the pore development, catalytic activity, and durability.^32^ The pore properties and textural structure of γ-Fe_2_O_3_@N-doped C_X_-600 were explored by N_2_ adsorption–desorption measurements. As shown in Figure 2a, typical type-IV isotherms with well-defined H4 hysteresis loops are found, implying the existence of mesoporous structures.^33^ Meanwhile, the nitrogen adsorption in the lower pressure region can be found, which indicates the presence of micropores. The pore size distributions (Figure 2b) of γ-Fe_2_O_3_@N-doped C_X_-600 obtained from the adsorption branch of the isotherms via the Barrett–Joyner–Halenda (BJH) method are centered at the pore size of ca. 3.8 nm. The γ-Fe_2_O_3_@N-doped C_L_ catalysts have a specific surface area of ca. 682 m^2^ g^–1^ (see Table 1), which is surpassing the specific surface area of γ-Fe_2_O_3_@N-doped C_C_-600 (362 m^2^ g^–1^) and γ-Fe_2_O_3_@N-doped C_S_-600 (527 m^2^ g^–1^). The above results indicate that the creation of hierarchical porosity may be attained by the KOH-activated carbonization. In other words, γ-Fe_2_O_3_@N-doped C_X_-600 dominantly exhibits a considerable portion of mesopores (0.21–0.26 cm^3^ g^–1^) with some microporosity. This result suggests that the synergetic effect of ball-milling and KOH activation promotes the development of mesoporosity, which is consistent with previous reports.^34,35^ The mesopore architecture benefits the electrolyte availability and electron transfer, leading to the boosted ORR activity. Among these biomass materials, the luffa and sugar cane bagasse are used as the precursors to produce the biocarbons (i.e., γ-Fe_2_O_3_@N-doped C_L_-600 and γ-Fe_2_O_3_@N-doped C_S_-600) with abundant micromesopores and higher specific surface areas, which may be served as promising catalyst supports for enhancing the ORR activity.

**Figure 2:**
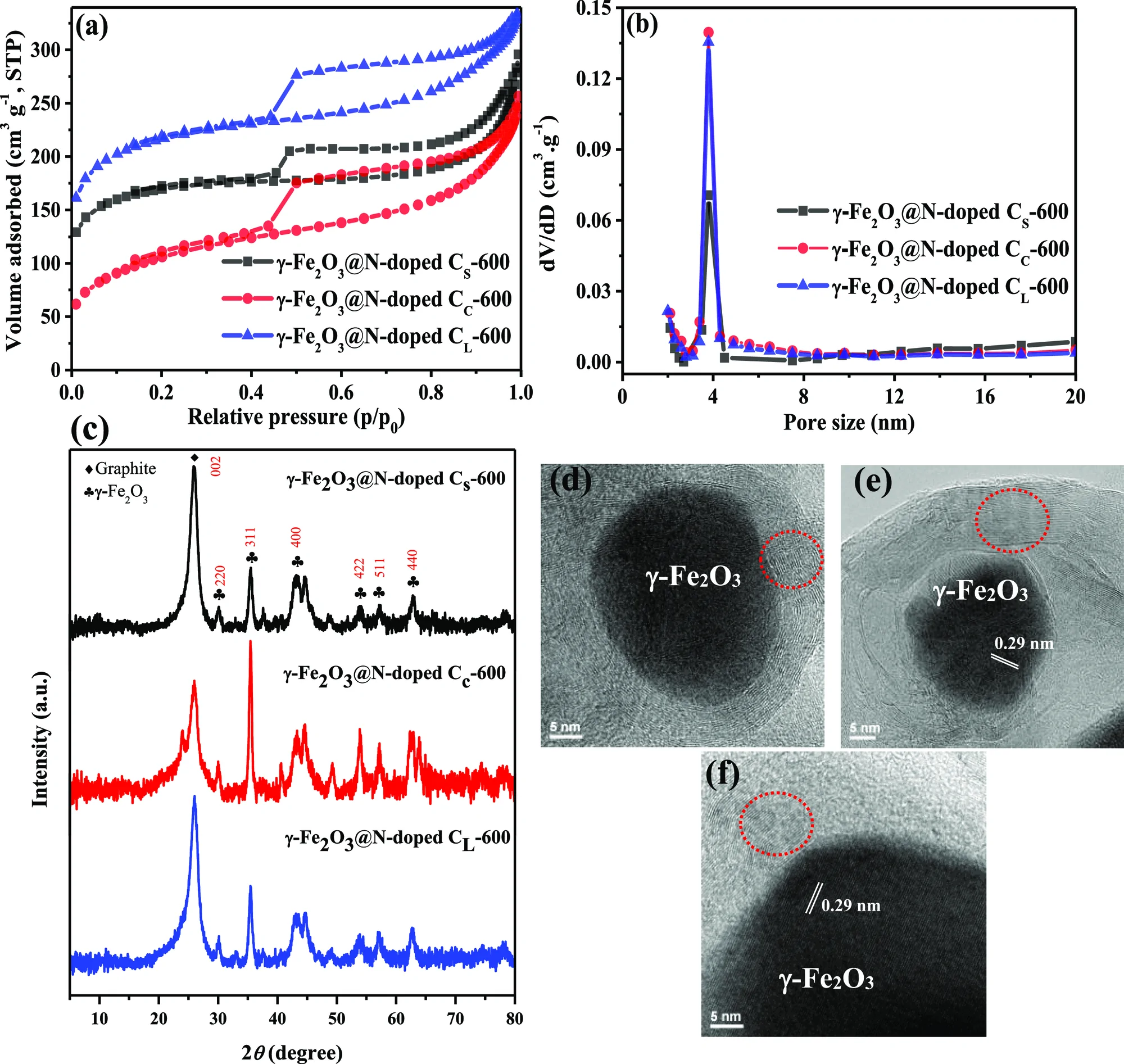
(a) N_2_ adsorption–desorption isotherms, (b) pore size distribution curves of γ-Fe_2_O_3_@N-doped C_X_-600, (c) XRD patterns of the as-prepared γ-Fe_2_O_3_@N-doped C_C_-600, and high-resolution transmission electron microscopy images of (d) γ-Fe_2_O_3_@N-doped C_S_-600, (e) γ-Fe_2_O_3_@N-doped C_C_-600, and (f) γ-Fe_2_O_3_@N-doped C_L_-600.

**Table 1:** Physical Properties of Various γ-Fe_2_O_3_@N-Doped C_X_-600

sample	Fe^a^ (wt %)	*S*_BET_ ^b^ (m^2^ g^–1^)	*V*_total_ ^c^ (cm^3^ g^–1^)	*V*_meso_ ^d^ (cm^3^ g^–1^)	*D* ^e^ (nm)
γ-Fe_2_O_3_@N-doped C_S_-600	12.5	527	0.46	0.26	3.48
γ-Fe_2_O_3_@N-doped C_C_-600	13.0	362	0.40	0.21	4.38
γ-Fe_2_O_3_@N-doped C_L_-600	12.8	682	0.52	0.26	3.03

a Loading amounts were measured by ICP-OES.

b BET specific surface area.

c Total pore volume.

d Mesopore volume.

e Average pore diameter.

The crystal structures of γ-Fe_2_O_3_@N-doped C_X_-600 were inspected by means of XRD. As shown in Figure 2c, the very intense and narrow peaks at around 26° correspond to the diffraction of graphite (002) crystalline planes, indicating the occurrence of higher graphitization degree.^6^ The diffraction peaks observed at 2θ = 30.2, 35.7, 43.3, 53.8, 57.1, and 63.0° can be, respectively, ascribed to the (220), (311), (400), (422), (511), and (440) crystal planes of maghemite (γ-Fe_2_O_3_) with a lattice constant of ca. 0.8350 nm.^20^ The other minor diffraction peaks could be assigned to the Fe_3_C phase.^36^ As reported earlier,^3^ the Fe metals can be incorporated onto carbon supports to generate iron carbide species due to the anchoring interaction. Since γ-Fe_2_O_3_ was identified as the predominant Fe-containing crystalline phase and only weak diffraction peaks attributable to Fe_3_C were observed, the ORR activity (discussed later) is expected to be primarily governed by γ-Fe_2_O_3_. The morphologies and structure of γ-Fe_2_O_3_@N-doped C_X_-600 were further examined by high-resolution TEM measurements. As shown in Figure 2d–f, the γ-Fe_2_O_3_ nanoparticles are covered with well-crystalline graphitic layers of carbons. The particle sizes of γ-Fe_2_O_3_ observed for the prepared catalysts follow the order γ-Fe_2_O_3_@N-doped C_L_-600 > γ-Fe_2_O_3_@N-doped C_C_-600 > γ-Fe_2_O_3_@N-doped C_S_-600. The lattice spacings observed for these nanoparticles are ca. 0.29 nm which can be ascribed to the (220) plane of γ-Fe_2_O_3_. In addition, graphitized carbon structure with a lattice spacing of 0.34 nm is found in the outer layer of γ-Fe_2_O_3_. The TEM result matches well with the above XRD data.^36^ In addition, scanning electron microscopy (SEM) and energy-dispersive X-ray spectroscopy (EDX) mapping images show that Fe, O, and N elements were uniformly dispersed with a Fe/O atomic ratio of ca. 2.0/3.4, suggesting the formation of Fe_2_O_3_.^37^

XPS spectra were additionally used to examine the surface compositions and bonding structure of catalysts. As shown in Figure 3a, all the C 1s XPS spectra display a main feature at ca. 284.5 eV (C=C), which is typically generated via sp^2^ hybridization. Moreover, the peak at ca. 285.6 eV is attributed to C–N bonds, indicating the successful doping of N atoms into the biocarbons. As reported earlier,^38^ the pyrrolic-N is ascribed to the N atoms bonding with two carbon atoms in the 5-membered heterocyclic rings that can provide two p-electrons to the π-conjugated system. The graphitic-N corresponds to the N atoms which are bonded to the carbon atoms in a graphene basal plane. Pyridinic-N is attributed to the N atoms binding with two carbon atoms on the edge of graphene planes.^39^ The XPS spectra at the N 1s observed for γ-Fe_2_O_3_@N-doped C_X_-600 are deconvoluted into four features, including pyridinic-N (398.6 ± 0.3 eV), pyrrolic-N (400.5 ± 0.3 eV), graphitic-N (401.2 ± 0.3 eV), and pyridinic-N-oxide (403.1 ± 0.1 eV), as shown in Figure 3b. It can be clearly seen that nitrogen doping onto biocarbons is successful, and thus, four different types of N-doping species (i.e., pyridinic-N, graphitic-N, pyrrolic-N, and pyridine-N-oxide) can be observed. Accordingly, the binding energies and atomic concentrations of N observed for γ-Fe_2_O_3_@N-doped C_X_-600 are depicted in Table 2. As mentioned previously,^40^ the pyridinic-N and graphitic-N could decrease the thermodynamic barrier and promote the kinetics of ORR, respectively. In addition, some researchers^41^ reported that the onset potential and limiting current density could be, respectively, improved by pyridinic-N and graphitic-N. However, the pyrrolic-N or pyridinic-N-oxide exhibit little effect on the ORR performance. It can be observed that γ-Fe_2_O_3_@N-doped C_C_-600 and γ-Fe_2_O_3_@N-doped C_S_-600 catalysts contain relatively higher proportions of pyridinic and graphitic nitrogen, which may play a crucial role for the ORR performances. In Figure 3c, the peaks observed for Fe species in the high-resolution XPS spectra are weak, indicating that the Fe elements are mostly confined by porous carbon shells. As such, the speciation of Fe could be barely observed by using XPS. The results are in good agreement with the aforementioned TEM and BET data. It is worthy to note that γ-Fe_2_O_3_ nanoparticles were encapsulated by a thin porous carbon layer (thickness = ca. 5–10 nm) with a mesopore ratio of 50–56%, ensuring sufficient exposure of active sites to the electrode–electrolyte–O_2_ interface. The ORR performance of γ-Fe_2_O_3_@N-doped C_X_-600 was assessed by means of RDE measurement in an O_2_-saturated 0.1 M KOH. As observed in Figures 4a and 1b, all the sample showed a steep reduction current between 0.8 and 1.0 V vs RHE. Among these γ-Fe_2_O_3_@N-doped C_X_-600 catalysts, γ-Fe_2_O_3_@N-doped C_S_-600 exhibits the better ORR activity (*E*_onset_ and *E*_1/2_ = 1.01 and 0.84 V vs RHE, respectively) which is comparable to commercially available Pt/C catalysts (JM-20, Johnson-Matthey 20 wt % Pt/Vulcan XC-72), as revealed by the more positive *E*_onset_ and *E*_1/2_ and highest limiting current density (see Table 3). In addition, the Tafel plots (Figures 4b and 1c) derived from polarization curves were employed to further investigate the intrinsic reaction kinetics for ORR. As a result, compared to γ-Fe_2_O_3_@N-doped C_C_-600 and γ-Fe_2_O_3_@N-doped C_L_-600, γ-Fe_2_O_3_@N-doped C_S_-600 exhibits a smaller Tafel slope of 80 mV dec^–1^, which is comparable to that of Pt/C catalysts (74 mV dec^–1^). The decrease in the Tafel slope from 109 mV dec^–1^ (γ-Fe_2_O_3_@N-doped C_C_-600) to 80 mV dec^–1^ (γ-Fe_2_O_3_@N-doped C_S_-600) indicated enhanced ORR kinetics. While the ORR on γ-Fe_2_O_3_@N-doped C_C_-600 was primarily limited by the first electron-transfer step, the lower Tafel slope of γ-Fe_2_O_3_@N-doped C_S_-600 suggested that this step was facilitated, and the reaction rate was further influenced by the adsorption of intermediates.^42^ The above results confirm that the higher transfer coefficient and faster kinetic of γ-Fe_2_O_3_@N-doped C_S_-600 for ORR can be achieved. As shown in Figure S2, the CV curves of various γ-Fe_2_O_3_@N-doped C_X_-600 were recorded at scan rates ranging from 20 to 70 mV s^–1^ in N_2_-saturated 0.1 M KOH. Among the prepared samples, γ-Fe_2_O_3_@N-doped C_S_-600 exhibited the highest electrochemical double-layer capacitance (*C*_dl_) of 2.0 mF cm^–2^ in the non-Faradaic potential region (Figure 4c) between 1.1 and 1.5 V (vs. RHE), where *C*_dl_ is proportional to the electrochemical active surface area.^29^ In view of the similar γ-Fe_2_O_3_ and N-doping amounts of γ-Fe_2_O_3_@N-doped C_X_-600, the boosted ORR behavior of γ-Fe_2_O_3_@N-doped C_S_-600 could be credited to the optimization of the mesoporous structure, the concentrations of pyridinic-N/graphitic-N, as well as smaller and well-dispersed γ-Fe_2_O_3_ nanoparticles which may be favorable for the oxygen diffusion and the exposure toward active sites. In terms of γ-Fe_2_O_3_@N-doped C_C_-600, even though the more pyridinic-N/graphitic-N amounts are observed, the limited active surface area leads to the inferior ORR activity. Likewise, γ-Fe_2_O_3_@N-doped C_L_-600 exhibits lower amounts of N-doped active sites with the higher surface area, leading to the worse ORR activity. Therefore, the above results can be ascribed to the synergistic contribution of pore structure with high surface area and speciations of nitrogen-doping, which could improve mass transport of reactants toward active sites, products, and the electrolyte, and thus exhibit better ORR performance.^10,14^ Subsequently, the ORR kinetics on the γ-Fe_2_O_3_@N-doped C_X_-600 samples was further investigated by using the K–L plots (*j* ^–1/2^ vs ω^–1/2^) at different potentials. Figures 4d,e and 1f also show the corresponding K–L plots which were further used to study electron transfer number (*n*, see Table 3). As a result, the *n* values of γ-Fe_2_O_3_@N-doped C_X_-600 catalysts are approximately estimated to be ca. 4. Moreover, the *n* values and H_2_O_2_ generated during ORR were measured by using RRDE (see Figures 1d and 4e). Consequently, the ORR of γ-Fe_2_O_3_@N-doped C_X_-600 catalysts can be proceeded with a 4-electron route, meaning the complete reduction of oxygen into water. Based on the XRD, TEM, and electrochemical ORR results together with insights from previous studies,^43,44^ a tentative mechanism could be proposed to explain the observed 4-electron process. First, the formation of FeOOH in the aqueous solution via the insertion of a proton into γ-Fe_2_O_3_ was observed (eq 5). Then, 2 mol of FeOOH could adsorb dissolved O_2_ (1 mol) to generate FeOOH–O_2,ads_–HOOFe via intermolecular hydrogen bonding (eq 6). Afterward, the O–O bonding of FeOOH–O_2,ads_–HOOFe was broken down to form active FeOOH–O^•^ species (eq 7). At last, the FeOOH–O^•^ species were reduced by an electron, and γ-Fe_2_O_3_ was recovered (eq 8).

5 $$2{Fe}_{2}O_{3}+2H_{2}O+2e^{-}↔2FeOOH+2{OH}^{-}+2FeO$$

6 $$2FeOOH+O_{2}↔FeOOH-O_{2,ads}-HOOFe$$

7 $$FeO+FeOOH-O_{2,ads}-HOOFe+e^{-}\to FeOOH-O^{\cdot }+{OH}^{-}+{Fe}_{2}O_{3}$$

8 $$FeO+FeOOH-O^{\cdot }+e^{-}↔{Fe}_{2}O_{3}+{OH}^{-}$$

**Figure 3:**
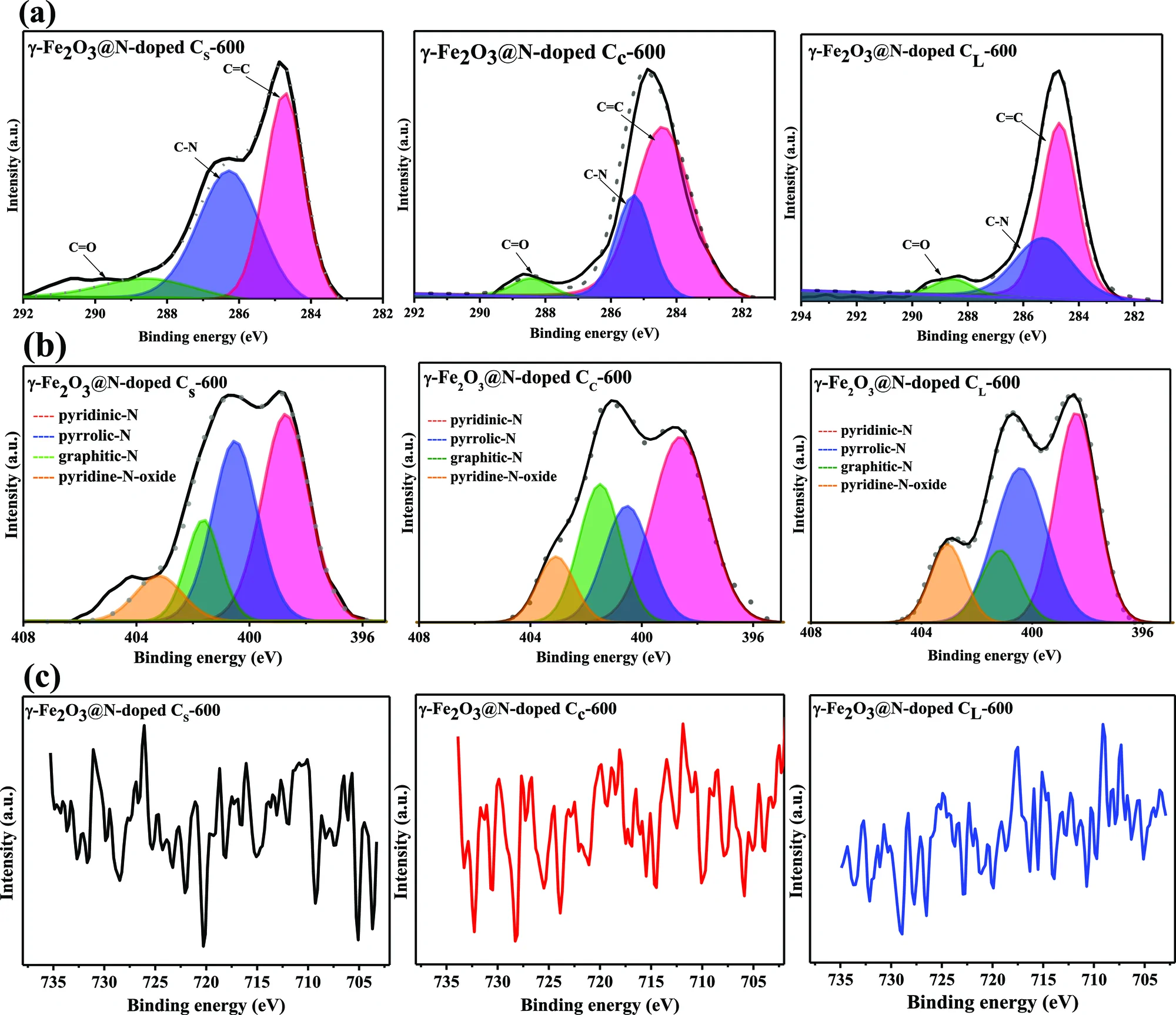
XPS spectra of γ-Fe_2_O_3_@N-doped C_X_-600 at the (a) C 1s, (b) N 1s, and (c) Fe 2p regions.

**Table 2:** Binding Energies and Relative Surface Concentrations of Nitrogen Species Obtained by Fitting the N 1s Core Level XPS Spectra

sample	species	binding energy (eV)	relative intensity (%)
γ-Fe_2_O_3_@N-doped C_S_-600	pyridinic-N	401.5	20.6
	pyrrolic-N	398.7	39.2
	graphitic-N	400.4	30.1
	pyridine-N-oxide	403.2	10.1
γ-Fe_2_O_3_@N-doped C_C_-600	pyridinic-N	398.7	43.4
	pyrrolic-N	400.5	22.1
	graphitic-N	401.5	24.2
	pyridine-N-oxide	403.1	10.3
γ-Fe_2_O_3_@N-doped C_L_-600	pyridinic-N	398.4	39.8
	pyrrolic-N	400.4	35.3
	graphitic-N	401.2	12.6
	pyridine-N-oxide	403.1	12.3

**Figure 4:**
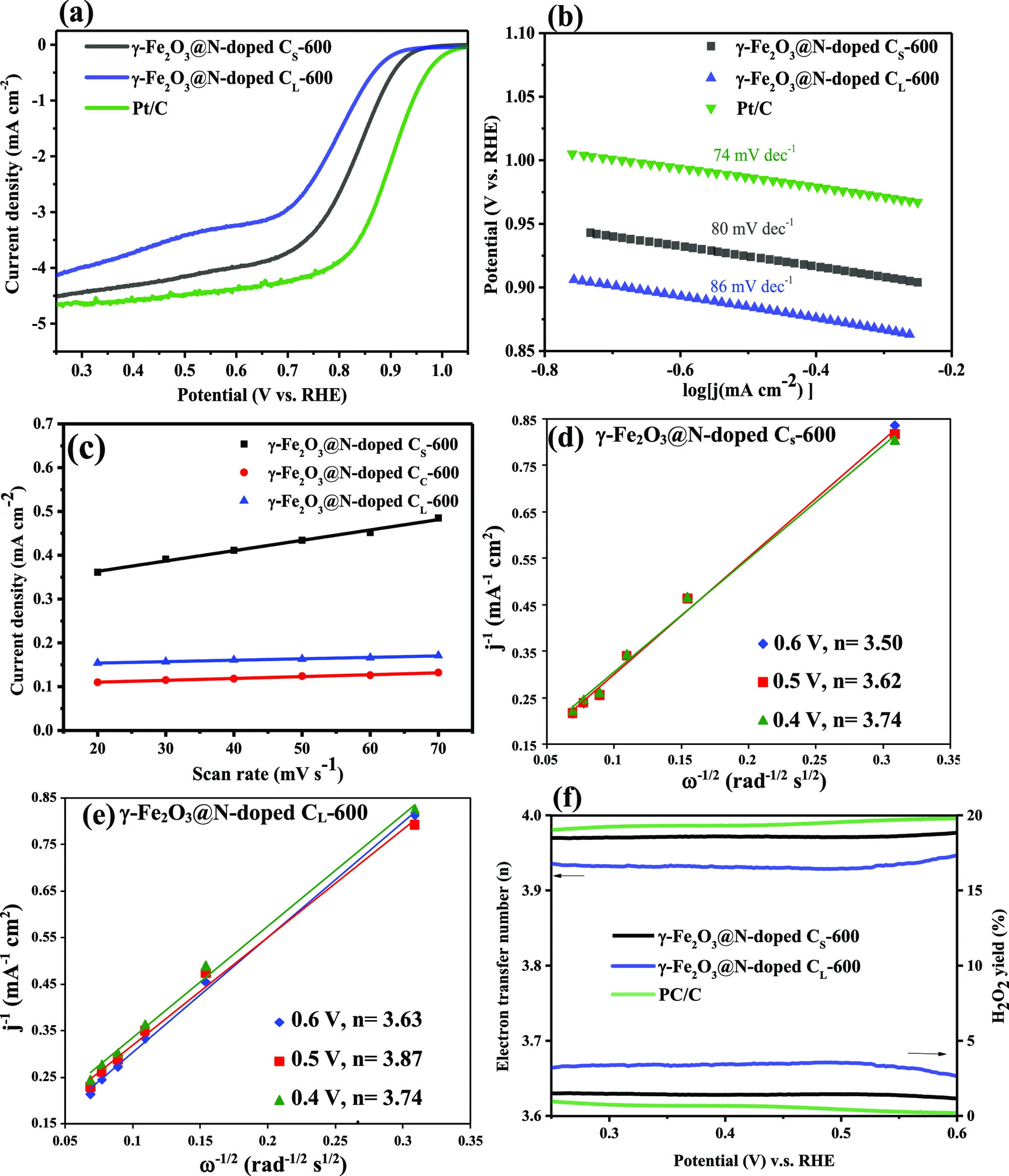
(a) LSV polarization curves, (b) Tafel plots, (c) *C*_dl_ at 1.3 V vs RHE, (d, e) Koutecky–Levich plots, and (f) H_2_O_2_ yield (%) and electron transfer number for O_2_ reduction on various γ-Fe_2_O_3_@N-doped C_X_-600 in O_2_-saturated 0.1 M KOH at a scan rate of 5 mV s^–1^ and different potentials, respectively.

**Table 3:** Electrochemical Parameters of Various γ-Fe_2_O_3_@N-Doped C_X_ and Commercial Pt/C during ORR

sample	*E*_onset_ (V)^a^	*I* (mA cm^–2^)^b^	*E*_1/2_ (V)^c^	*n* ^d^
γ-Fe_2_O_3_@N-doped C_S_-600	1.01	4.5	0.84	3.6
γ-Fe_2_O_3_@N-doped C_C_-600	0.91	3.8	0.78	3.7
γ-Fe_2_O_3_@N-doped C_L_-600	0.97	4.2	0.81	3.7
Pt/C	1.04	4.7	0.90	3.8

a ORR onset potential (vs RHE).

b Limiting current density.

c Half-wave potential.

d The numbers of electrons transferred.

The preparation of durable ORR catalysts is also a crucial concern for practical applications. Accordingly, the cyclic ORR and methanol-resistance tests were carried out. In Figure 5, γ-Fe_2_O_3_@N-doped C_S_-600 shows the better stability (i.e., only a small *E*_1/2_ reduction of ca. 7 mV) toward ORR after 2000 cycles of operation. In addition, the prepared catalysts exhibit superior tolerance toward methanol crossover in the direct-methanol fuel cells as compared to commercial Pt/C catalysts. These results obtained from electrochemical measurements have demonstrated that the high surface area biocarbon supports with hierarchical porosity and high density of N-doped surface functionalities can provide accessible active sites for durable ORR.

**Figure 5:**
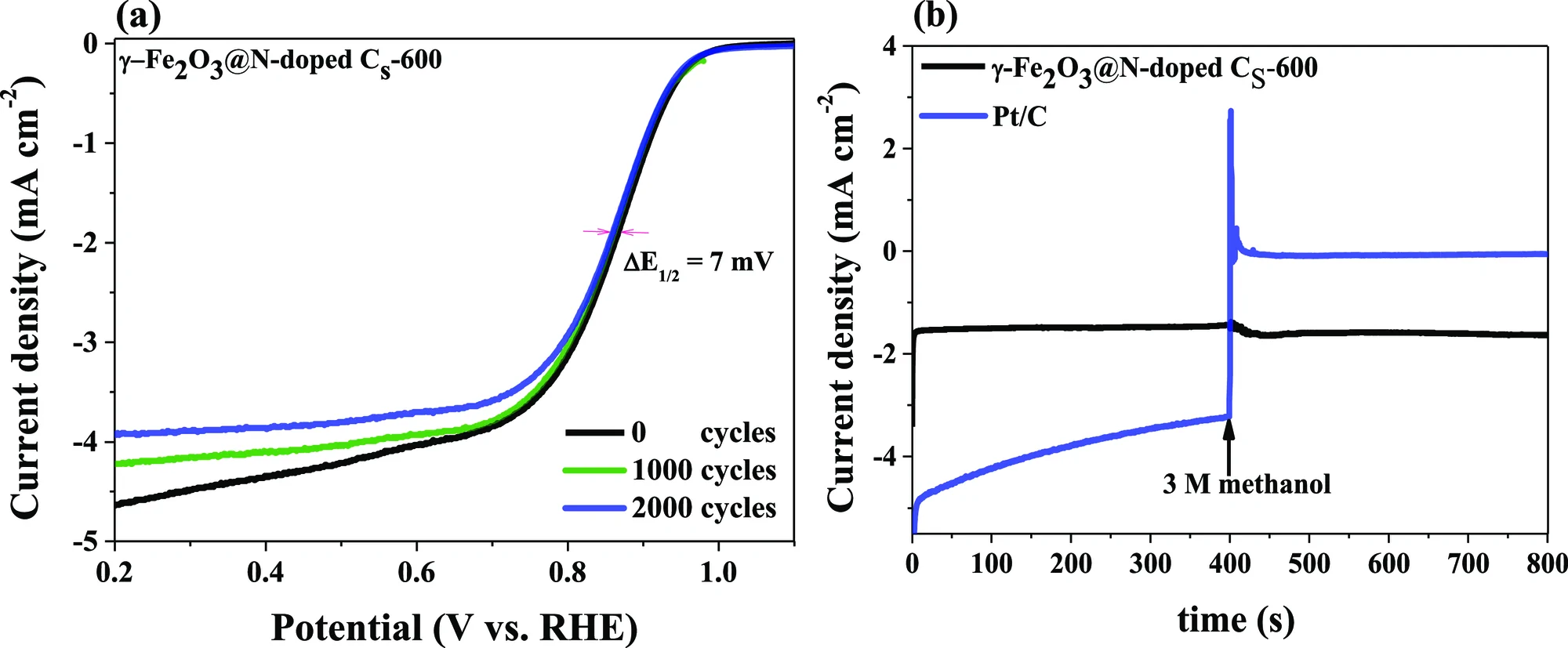
(a) LSV curves of γ-Fe_2_O_3_@N-doped C_S_-600 for ORR in an O_2_-saturated 0.1 M KOH solution at room temperature after various cycles (potential range: 0.2–1.1 V vs RHE, rotation speed: 1600 rpm, and scan rate: 5 mV s^–1^) and (b) chronoamperometric curves of γ-Fe_2_O_3_@N-doped C_S_-600 and Pt/C in an O_2_-saturated 0.1 M KOH electrolyte with the rotation rate of 1600 rpm at 0.6 V vs RHE; 3 M methanol was added at around 400 s.

To compare previously reported Fe_2_O_3_-supported carbons with the prepared γ-Fe_2_O_3_@N-doped C_S_-600 for ORR, some related works and their corresponding discussions are summarized below. Gan et al.^19^ synthesized the Fe_2_O_3_-encapsulated and Fe–N_*x*_-containing porous carbon spheres (Fe_2_O_3_/N-MCCS) by using a complicated process (i.e., polymerization, carbonization, silica etching, Fe­(NO_3_)_3_ loading, and NH_3_ treatments). The resultant Fe_2_O_3_/N-MCCS had the *E*_onset_ and *E*_1/2_ values of 0.965 and 0.837 V vs RHE, respectively, which were worse than those of γ-Fe_2_O_3_@N-doped C_S_-600. Gopal et al.^20^ loaded Fe_2_O_3_ on various carbons (i.e., Ketjen Black (KB), Vulcan Carbon XC 72R (VC), and graphene oxide). As a result, the ORR activity of Fe_2_O_3_/KB was the best (*E*_onset_ = 0.84 eV vs RHE). Wang et al.^21^ reported a direct annealing method to fabricate the N-doped hierarchically porous carbon nanospheres with Fe_3_O_4_/Fe_2_O_3_/Fe nanoparticles (Fe-CNSs-N), which exhibited the *E*_onset_ and *E*_1/2_ values of 0.948 and 0.835 V vs RHE, respectively. Miao et al.^44^ reported the synthesis of γ-Fe_2_O_3_ supported on N-doped carbons derived from wood biowastes, and their corresponding *E*_onset_ and *E*_1/2_ values were 0.98 and 0.78 V vs RHE. Ren et al.^45^ demonstrated that the in-site formation of Fe–N_4_ single-atom catalytic sites assisted with Fe_2_O_3_ species which were abundant oxygen vacancies by the pyrolysis of an iron porphyrin conjugated mesoporous polymer. The resultant electrocatalysts exhibited *E*_onset_ and *E*_1/2_ values of 0.987 and 0.901 V vs RHE, respectively. Zhang et al.^46^ synthesized the Fe_2_O_3_ nanoparticles decorated on N-doped mesoporous carbons derived from mulberry leaves by using the cost-effective and simple route. The *E*_onset_ and *E*_1/2_ values were found to be 0.936 and 0.776 V vs RHE, respectively. As can be seen form the above results, our prepared γ-Fe_2_O_3_@N-doped C_S_-600 (*E*_onset_ and *E*_1/2_ values = 1.01 and 0.84 V vs RHE) almost surpassed the aforementioned ORR catalysts. In addition, the ORR performance of various biocarbons derived from biomass is listed in Table 4. The prepared γ-Fe_2_O_3_@N-doped C_S_-600 obtained from sugar cane bagasse via a facile and simple ball-milling route exhibits the superior activity toward ORR as compared to the earlier reported biocarbon electrocatalysts. It is noteworthy that this is the first report on facile synthesis of core–shell γ-Fe_2_O_3_ nanoparticles@N-doped mesoporous carbons derived from biomass which possess superior electrochemical properties during ORR.

**Table 4:** Comparison of ORR Performance among Previously Reported Biocarbons

catalysts	biomass precursors	*E*_onset_ ^a^	*E*_1/2_ ^b^	*J*_L_ ^c^	reference
HC-900	lotus leaves	0.95	0.84	4.3	^47^
AC-F-U-P	coconut shells	0.95	0.76	5.3	^48^
SCup	seaweeds	0.96	0.82	4.5	^49^
NPCN	Gingko leaves	0.93	0.82	5.5	^50^
Fe_3_C(Fe)@NDGL	glucose	0.91	0.82	6.0	^51^
HNORI-M-AT	Nori algae	0.98	0.83	5.8	^52^
NDPG	eggplant	0.93	0.63	5.5	^53^
NPAC_Co_	Pomelo peels	0.88	0.79	5.2	^54^
HAZ-800	bamboo fungus	1.06	0.90	3.6	^55^
SKPHD	Sargassum	0.79	0.60	4.6	^56^
N-CNDs@MCNSs	grass	0.89	0.77	3.1	^57^
SC-FeCo-N@N-carbon	sugar cane bagasse	0.91	0.81	2.6	^16^
NBCW-900	Paulownia wood	0.970	0.849	5.11	^58^
γ-Fe_2_O_3_@N-doped C_S_-600	sugar cane bagasse	1.01	0.84	4.5	this study

a ORR onset potential (V vs. RHE).

b Half wave potential (V vs. RHE).

c Limiting current density (mA cm^-2^)

## Conclusions

In summary, the γ-Fe_2_O_3_@N-doped C_X_-600 catalysts which composed of active γ-Fe_2_O_3_ nanoparticles with N-doped graphitic mesoporous carbons were produced from the various biomass precursors via a combination of carbonization, activation with KOH, and ball-milling. Electrochemical tests demonstrated that the γ-Fe_2_O_3_@N-doped C_S_-600 catalysts derived from sugar cane bagasse possess the better performance toward ORR with a 4-electron transfer pathway which can be comparable to commercially available catalysts. In addition, the greater limiting current density, surpassing durability, and resistance to the effect of methanol crossover were also observed. The promoted ORR can be accredited to abundant amounts of active N speciations, large surface areas with hierarchical porous structures, and active γ-Fe_2_O_3_. More importantly, the γ-Fe_2_O_3_@N-doped C_X_-600 catalysts can be prepared by using abundant biomass and cheap chemicals such as melamine and iron salts as starting precursors. This study provides one of the alternative methods to develop the cost-effective non-noble metal electrodes for practical fuel cell applications.

## Supplementary Material



## Notes

SEM and EDX mapping of γ-Fe_2_O_3_@N-doped C_S_-600 and CV curves of γ-Fe_2_O_3_@N-doped C_X_-600 at different scan rates (20–70 mV s^–1^) in N_2_-saturated 0.1 M KOH (PDF)
