# Supplementary material for: Mechanochemical Synthesis of γ‑Fe2O3 Nanoparticles@N-Doped Mesoporous Carbons Derived from Biomass for Oxygen Reduction
Source: ACS Omega. 2026 Aug 10;11(33):50082–92. doi: 10.1021/acsomega.6c05376 (PMC13520441; doi:10.1021/acsomega.6c05376)
Supplement: Supplementary file 1 [file ao6c05376_si_001.pdf]

Supporting Information

Mechanochemical synthesis of γ-Fe_2O_3 nanoparticles@N-doped mesoporous carbons derived from biomass for oxygen reduction

Hung-Ta Yu, Ling-Wei Wei and Shou-Heng Liu*

Department of Environmental Engineering, National Cheng Kung University, Tainan 70101, Taiwan

*Corresponding author. *E-mail address*: shliu@mail.ncku.edu.tw (S.-H. Liu)

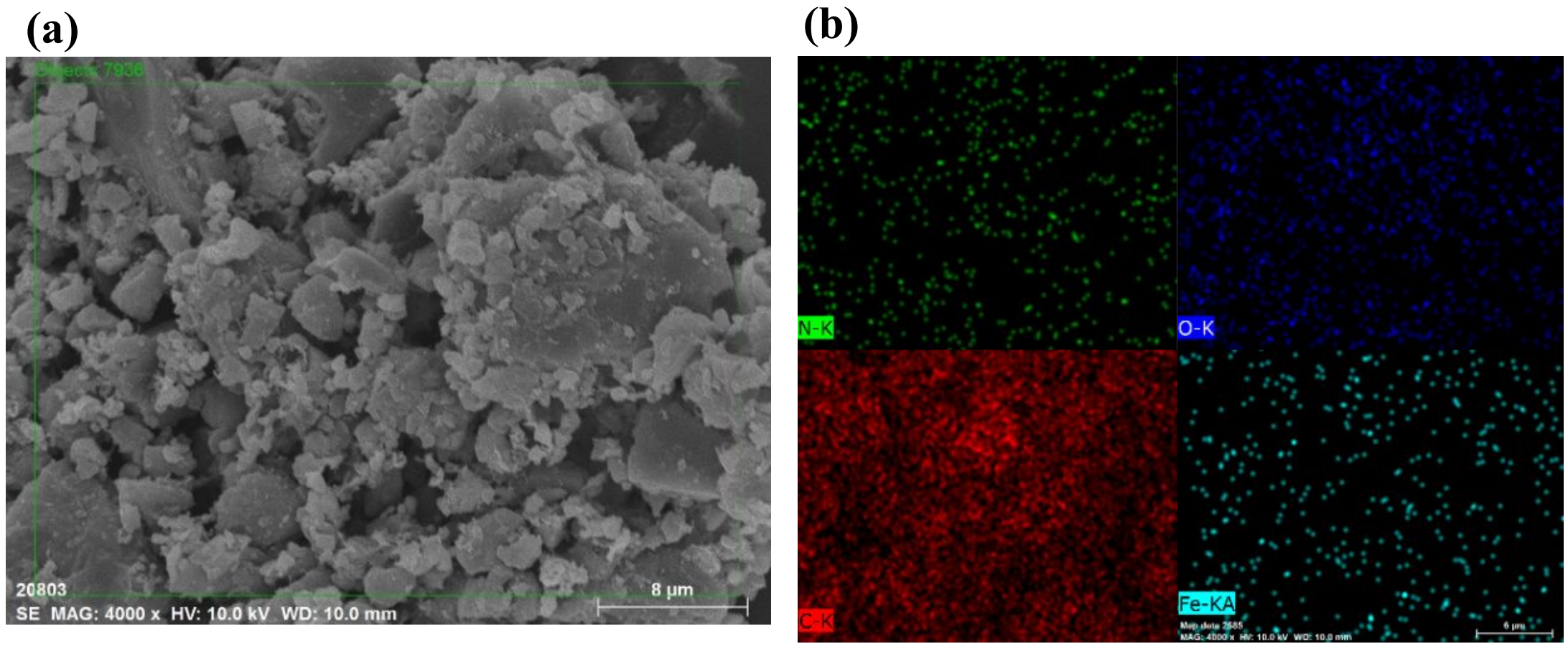

Figure S1. (a) SEM and (b) EDX mapping of γ-Fe_2O_3@N-doped C_S-600.

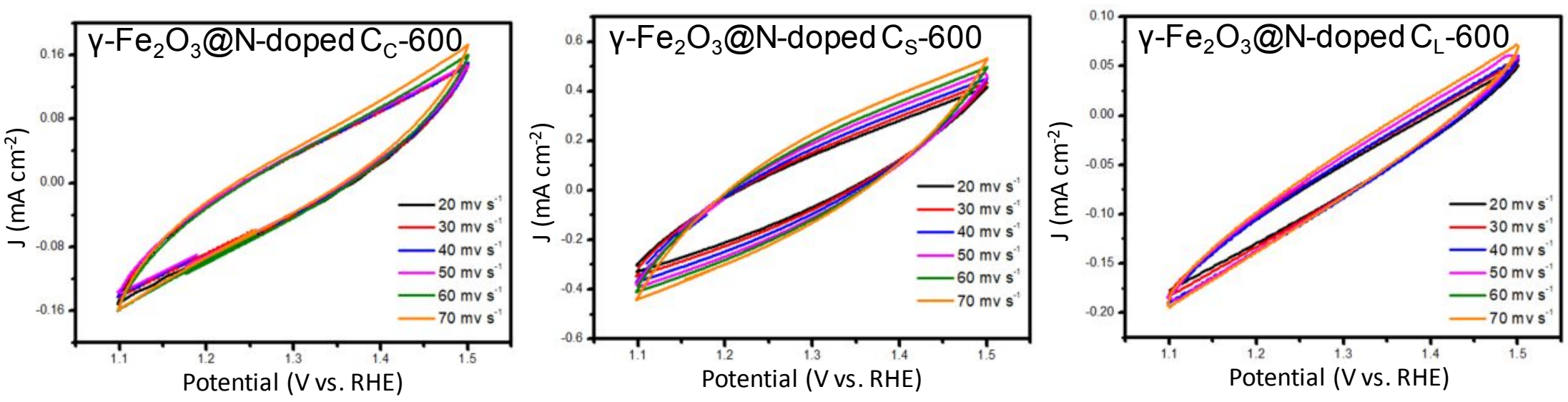

Figure S2. CV curves of γ-Fe_2O_3@N-doped C_X-600 at different scan rates (20-70 mV s^{-1}) in N_2-saturated 0.1 M KOH.
